# Human judgement forecasting of COVID-19 in the UK

**DOI:** 10.12688/wellcomeopenres.19380.1

**Published:** 2023-09-19

**Authors:** Nikos I. Bosse, Sam Abbott, Johannes Bracher, Edwin van Leeuwen, Anne Cori, Sebastian Funk

**Affiliations:** 1 Department of Infectious Disease Epidemiology, London School of Hygiene & Tropical Medicine, London, WC1E 7HT, UK; 2 NIHR Health Protection Research Unit in Modelling & Health Economics, London, UK; 3 Computational Statistics Group, Heidelberg Institute for Theoretical Studies, Heidelberg, Germany; 4 Chair of Statistical Methods and Econometrics, Karlsruhe Institute of Technology, Karlsruhe, Germany; 5 Modelling Economics Unit, UK Health Security Agency, London, UK; 6 MRC Centre for Outbreak Analysis and Modelling, Department of Infectious Disease Epidemiology, School of Public Health, Imperial College London, London, England, UK

**Keywords:** forecasting, human judgement forecasting, COVID-19, UK, United Kingdom, Weighted Interval Score

## Abstract

**Background** In the past, two studies found ensembles of human judgement forecasts of COVID-19 to show predictive performance comparable to ensembles of computational models, at least when predicting case incidences. We present a follow-up to a study conducted in Germany and Poland and investigate a novel joint approach to combine human judgement and epidemiological modelling.

**Methods** From May 24th to August 16th, 2021, we elicited weekly one to four week ahead forecasts of cases and deaths from COVID-19 in the UK from a crowd of human forecasters. A median ensemble of all forecasts was submitted to the European Forecast Hub. Participants could use two distinct interfaces: in one, forecasters submitted a predictive distribution directly, in the other forecasters instead submitted a forecast of the effective reproduction number *R _t_*. This was then used to forecast cases and deaths using simulation methods from the EpiNow2 R package. Forecasts were scored using the weighted interval score on the original forecasts, as well as after applying the natural logarithm to both forecasts and observations.

**Results** The ensemble of human forecasters overall performed comparably to the official European Forecast Hub ensemble on both cases and deaths, although results were sensitive to changes in details of the evaluation. *R _t_* forecasts performed comparably to direct forecasts on cases, but worse on deaths. Self-identified “experts” tended to be better calibrated than “non-experts” for cases, but not for deaths.

**Conclusions** Human judgement forecasts and computational models can produce forecasts of similar quality for infectious disease such as COVID-19. The results of forecast evaluations can change depending on what metrics are chosen and judgement on what does or doesn’t constitute a “good” forecast is dependent on the forecast consumer. Combinations of human and computational forecasts hold potential but present real-world challenges that need to be solved.

## Introduction

Infectious disease modelling and forecasting has attracted wide-spread attention during the COVID-19 pandemic and helped inform decision making in public health organisations and governments ^1, 2^. Most forecasts used to inform decision making were based on computational models of COVID-19, but some authors also explored human judgement forecasting as an alternative or in combination ^3– 6^.

Past research found that in the context of infectious disease forecasting, human judgement forecasts could achieve predictive performance broadly comparable to forecasts generated based on mathematical modelling, in particular when forecasting incident cases, rather than lagged indicators indicators like deaths. Farrow *et al.* ^7^ found that an aggregate of human predictions outperformed computational models when predicting the 2014/15 and 2015/16 flu season in the US. However, a comparable approach performed worse than computational models at predicting the 2014/15 outbreak of chikungunya in the Americas. Bosse *et al.* ^3^ found an ensemble of human forecasters to outperform an ensemble of computational models when predicting cases of COVID-19 in Germany and Poland, but performing worse when predicting incident deaths. Similarly, McAndrew *et al.* ^5^ reported an ensemble of human forecasters to perform comparably to an ensemble of computational models when predicting incident COVID-19 cases, and worse when predicting incident deaths. Farrow *et al.* ^7^ and in particular Bosse *et al.* ^3^ struggled to recruit many participants (numbers of active forecasters ranged from 22 to 61 in McAndrew *et al.* ^5^, 7 to 24 in Farrow *et al.* ^7^, and 4 to 10 in Bosse *et al.* ^3^). It is important to note that in previous studies (and also this one) human forecasters were free to use any resources, including computational models, in the process of creating a forecast, making it difficult to completely separate human judgement and computational modelling.

In some situations, human judgement forecasting may have advantages relative to computational models. Human judgment may be particularly useful to provide timely forecasts in situations where data is sparse and many parameters are hard to quantify. Humans are also generally able to answer a broad set of question (such as for example the likelihood that a given actor will take some specified action) and can take factors into account that are hard to encode in a computational model. On the other hand, human judgement forecasting is difficult to scale due to the time and effort required, and humans may be at a disadvantage at tasks that strongly benefit from the ability to perform complex computations. Also, the use of human judgement forecasts by decision makers may be complicated by the lack of clarity of the basis on which they were made.

Methods that aim to combine human judgement and mathematical modelling are therefore appealing, though we note that presenting this as a binary choice is misleading. Most computational models in use in epidemiology have at least some element of human judgement supporting their structure or usage. Also, human forecasters often make use of approaches such as calculating a base rate of incidences, or extrapolating current trends, which are in reality equivalent to simple models. One explicit method to combine separate human judgement and computational model forecasts with the goal of improving predictive performance is an ensemble. This has been shown to improve performance across model types ^5^. Farrow *et al.* ^7^, Bosse *et al.* ^3^, Swallow *et al.* ^8^ and others suggested additional possibilities in the context of infectious diseases that may also help reduce the amount of human effort required. One approach is to use human forecasts, for example of relevant disease parameters, as an input to computational modelling. Another approach is to use mathematical modelling in explicit combination with human judgement, for example by giving experts the option to make post-hoc adjustments to model outputs. Bosse *et al.* ^3^ proposed asking human forecasters to forecast the effective reproduction number *R _t_* (the average number of people an infected person would infect in turn) based on modelled estimates and to then use this forecast in a mathematical simulation model in order to obtain forecasts for observed case and death numbers.

This paper represents a follow-up study to Bosse *et al.* ^3^ in the United Kingdom with forecasts made over the course of thirteen weeks between May and August 2021. Forecasts were elicited from experts and laypeople as part of a public forecasting tournament, the "UK Crowd Forecasting Challenge", using a web application. All forecasts were submitted to the European COVID-19 Forecast Hub, one of several Forecast Hubs that have been systematically collating forecasts of different COVID-19 forecast targets in the US ^1^, Germany and Poland ^9, 10^, and Europe ^11^. This study aims to investigate whether the original findings in Bosse *et al.* ^3^ with respect to forecaster performance replicate in a different country, in a different time period, and with an increased number of participants. In addition, it explores the approach proposed in Bosse *et al.* ^3^ to ask participants for a forecast of the estimated effective reproduction number *R _t_* which is then translated into a forecast of cases and deaths using a simulation model. We describe this approach as human in the loop computational modelling and consider it a formalisation of often practiced manual intervention in computational forecasts.

## Methods

### Interaction with the European Forecast Hub

The European COVID-19 Forecast Hub ^11^ was launched in March 2021 in order to elicit weekly predictions for various COVID-19 related forecast targets from different research groups. The forecasts evaluated in this study were submitted every Monday before 11.59pm GMT between May 24 2021 and August 16 2021. Forecasts were made for incident weekly reported numbers of cases of and deaths from COVID-19 on a national level for various European countries over a one to four week forecast horizon. While forecasts were submitted on Mondays, weeks were defined as epidemiological weeks, ending on a Saturday, and starting on Sunday. Forecast horizons were therefore in fact 5, 12, 19 and 26 days. Submissions to the European Forecast Hub followed a quantile-based format with 23 quantiles of each output measure at levels 0.01, 0.025, 0.05, 0.10, 0.15,. . . , 0.95, 0.975, 0.99. Every week, forecasts submitted to the hub were automatically checked for conformity with the required format and all eligible forecasts combined into different ensembles. Until the 12th of July 2021 the default Hub ensemble ("EuroCOVIDhub-ensemble") shown on all official Forecast Hub visualisations ( https://covid19forecasthub.eu/) was a mean ensemble (i.e., the *α*-quantile of the ensemble is given by the mean of all submitted *α*-quantiles). From the 29th of July onwards, the default Forecast Hub ensemble became a median ensemble. The median number of models included in the Forecast Hub ensemble for the UK during the study period was 9 for cases and 10 for deaths (see Figure 1 in the SI).

**Figure 1.**
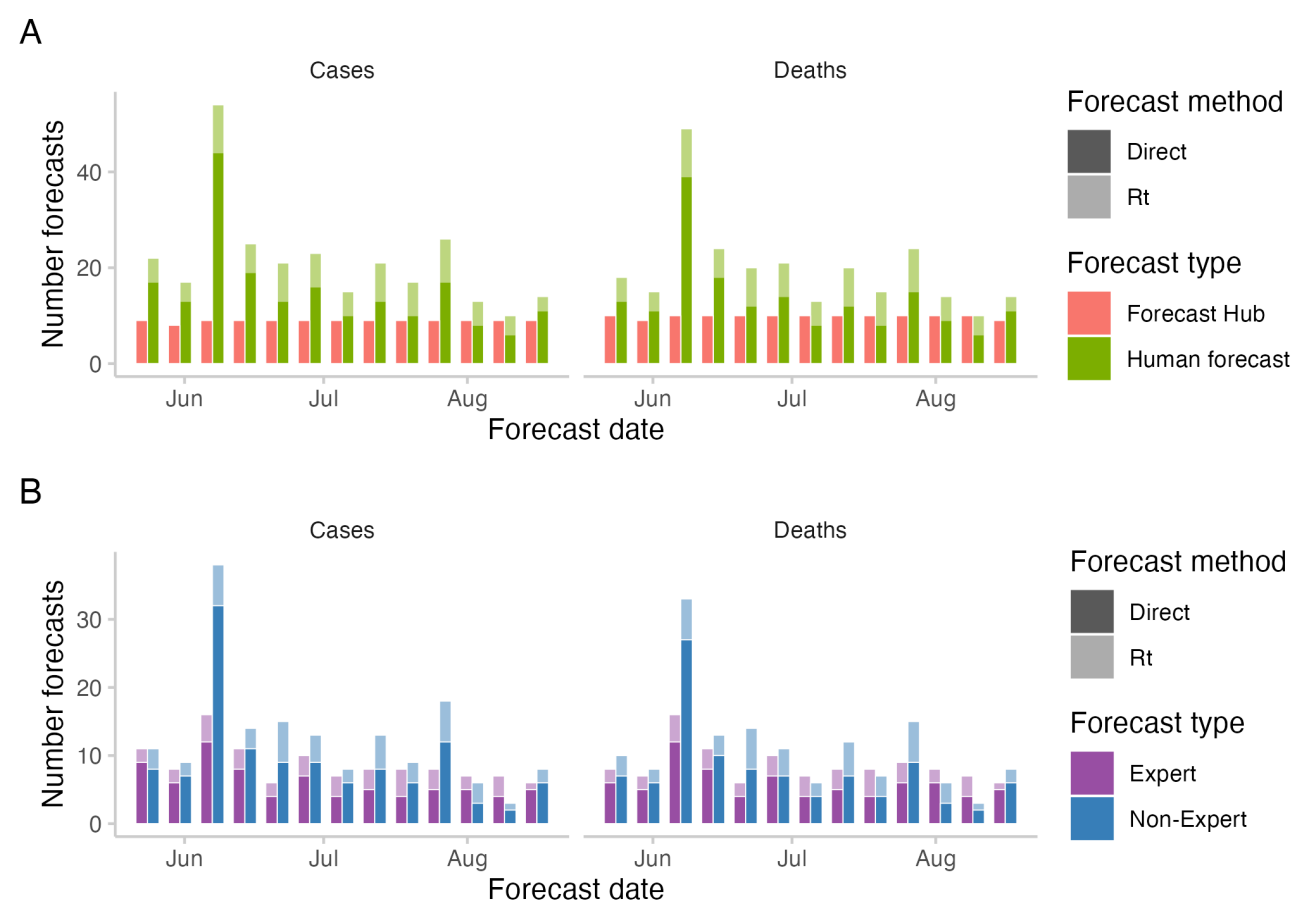
Number of forecasts across the study period. **A**: number of forecasts included in the Hub ensemble and the combined crowd ensemble. **B**: number of forecasts by "experts" and "non-experts". Expert status was determined based on the participant’s answer to the question whether they "worked in infectious disease modelling or had professional experience in any related field".

Ground-truth data on daily reported test positive cases and deaths linked to COVID-19 were provided by the European Forecast Hub and sourced from the Johns Hopkins University (JHU). Data were subject to reporting artifacts and revisions. All data points were marked as anomalous retrospectively by the European Forecast Hub if in subsequent updates data was changed by more than 5 percent. In August 2022 JHU switched the data source for their UK death numbers from "deaths within 28 days of a positive COVID test" to "Deaths with COVID-19 on the death certificate" and revised all their past data to guarantee consistency. The 2021 UK ground truth death data as it was made available through the European Forecast Hub in 2021 is therefore substantially different and on average lower than the data available as of early 2023. The data and revisions are displayed in Figure 2 in the Supplementary Information ^12^. All results presented here were derived based on the original data available in 2021, which were available through the European COVID-19 Forecast Hub GitHub repository ( https://github.com/covid19-forecast-hub-europe/covid19-forecast-hub-europe).

### Human judgement forecasts

Forecasts of incident cases and deaths linked to COVID-19 in the UK were elicited from individual participants every week through a web application ( https://cmmid-lshtm.shinyapps.io/crowd-forecast/) described in 3. The application is based on R ^13^ shiny ^14^ and is available as an R package called crowdforecastr ^15^. When signing up, participants could self-identify as "experts" if they worked in infectious disease modelling or had professional experience in any related field.

The web application offered participants two different ways of making a forecast, called ’direct’ (or ’classical’) and ’ *R _t_* forecast’. To make a ’direct’ forecast (as described in more detail in 3), participants selected a predictive distribution (by default a log-normal distribution) and adjusted the median and width of the distribution to change the central estimate and uncertainty at each forecast horizon.

Just as in the previous study, the default forecast shown was a repetition of the last known observation with constant uncertainty around it. The shown distribution was the exponential of a normal distribution with mean log(last value) and uncertainty set to the standard deviation of the last four changes in weekly log observed forecasts (i.e., as *σ*(log(value4) *−* log(value3), log(value3) *−* log(value2), . . . )). In addition to information about past observations, participants could see various metrics and data such as the test positivity rate and vaccination rate sourced from Our World in Data ^16^. Figure 3 in the Supplementary Information ^12^ shows a screenshot of the forecast interface for direct forecasts.

In addition to the ‘direct’ forecasts, we implemented a second forecasting method (‘ *R _t_* forecasts’), where we asked participants to make a forecast of the effective reproduction number *R _t_*. This forecast was made based on a baseline estimate produced by the EpiNow2 ^17^ R ^13^ package effective reproduction number model which we also used in 3 as a standalone computational model. The estimate produced by EpiNow2 was shown as the default forecast and could be adjusted by the user. The resulting *R _t_* forecast was then translated into a forecast of cases using the simulation model from the EpiNow2 R package, which implements a renewal equation based ^18^ generative process for latent infections. We chose a Gaussian Process prior with mean 0 for the first differences of the effective reproduction number in time, implying that in the absence of informative data the reproduction number would remain constant on average, with uncertainty increasing with the temporal distance to informative data points. Latent infections were convolved with delay distributions representing the incubation period and reporting delay, and assumed to follow a negative binomial observation model with a day of the week effect to produce an estimate of reported cases. This approach has been widely used for short-term forecasting ^3, 11^ and used to produce reproduction number estimates ^19– 21^. Further details are given in the Supplementary Information ^12^.

To obtain forecasts for deaths, we similarly fit a model that convolved observed and predicted reported cases as implied by the *R _t_* forecast over a delay distributions ^20, 21^ and scaled them by a fixed ratio to model the time between a case report and a reported death and the case fatality ratio using the EpiNow2 R package ^17^. Further details are given in the Supplementary Information ^12^.

As *R _t_*-estimates up to at least two weeks prior to the forecast data were uncertain due to their dependence on partially complete observations of underlying infections given the delays from infection to report, we also asked participants to submit an estimate of *R _t_* for the two weeks prior to the current forecast date. Participants were therefore asked to estimate/predict six *R _t_* values, four of them beyond the forecast horizon. In order to obtain sample trajectories needed as input for the simulation model, we drew 1000 samples from the six provided distributions. These samples were ordered and corresponding samples treated as one sample trajectory. Samples for daily values were obtained by linearly interpolating between weekly samples.

Upon pressing a button, participants could see a preview of the evolution of cases implied by their current *R _t_* forecast. However, due to lack of development time, participants could not preview the death forecast implied by their current input for *R _t_* nor could they influence the estimated case fatality ratio or delay between reported cases and reported deaths. Figure 4 in the Supplementary Information ^12^ shows a screenshot of the forecast interface for *R _t_* forecasts.

Every week, we submitted an ensemble of individual forecasts to the European Forecast Hub. In contrast to the ensemble of human forecasts described in Bosse *et al.* ^3^, we used the quantile-wise median, rather than the quantile-wise mean to combine predictions, drawing upon insights gained from the COVID-19 Forecast Hubs ^22^. We submitted three different ensembles to the Hub: The first one, "epiforecasts-EpiExpert_direct" (here called "direct crowd forecast" or "crowd-direct") was a quantile-wise median ensemble of all the direct forecasts. "epiforecasts-EpiExpert_Rt" (here called " *R _t_* forecast" or "crowd-rt") was a median ensemble of all forecasts made through the *R _t_* interface. "epiforecasts-EpiExpert" (here called "combined crowd ensemble" or "crowd-ensemble") was a median ensemble of all forecasts together. A participant could enter the combined crowd ensemble twice if they had submitted both a direct and an *R _t_* forecast. Before creating the ensemble, we deleted forecasts that were clearly the result of a user or software error (such as forecasts that were zero everywhere).

### The UK Crowd Forecasting Challenge

To boost participation compared to our last crowd forecasting study in Germany and Poland ^7^ which struggled in this regard, we announced an official tournament, the "UK Crowd Forecasting Challenge". Participants were asked to submit weekly predictions for reported cases and deaths linked to COVID-19 in the United Kingdom one to four weeks into the future. Everyone who had submitted a forecast for targets in the UK during the tournament period from the 24th of May 2021 to the 16th of August 2021 was deemed a participant and eligible for a prize. The first prize was 100 GBP, second prize 50 GBP and third prize 25 GBP. Participant performance was determined using the mean weighted interval score (WIS) on the log scale (see details in the next Section), averaged across forecast dates, horizons and forecast targets. For the tournament ranking, participants who did not submit a forecast in a given week were assigned the median score of all other participants who submitted a forecast that week. The UK crowd forecasting challenge was announced over Twitter and our networks. In addition, we created a project website, https://crowdforecastr.org, made weekly posts on Twitter and sent participants who had registered on the online application weekly emails with a reminder and a summary of their past performance. A public leaderboard was available on our website https://epiforecasts.io. Participants could choose to make a direct forecast as well as an *R _t_* forecast and were counted as two separate forecasters and eligible for prizes twice. Weekly forecasts had to be submitted between Sunday 12pm and Monday 8pm UK time.

### Analysis

We scored forecasts using the weighted interval score ^23^. For a single interval, the interval score is computed as


$$IS_{\alpha }(F,y)=(u-l)+\frac{2}{\alpha }\cdot (l-y)\cdot 1(y\leq l)+\frac{2}{\alpha }\cdot (y-u)\cdot 1(y\geq u),$$

where 1() is the indicator function, *y* is the true value, and *l* and *u* are the $\frac{\alpha }{2}$ and 1 − $\frac{\alpha }{2}$ quantiles of the predictive distribution *F*, i.e., the lower and upper bound of a single prediction interval. For a set of *K* prediction intervals and the median *m*, the score is computed as a weighted sum,


$$WIS=\frac{1}{K+0.5}\cdot \left(w_{0}\cdot \text{|}y-m\text{|}+\sum_{k=1}^{K}w_{k}\cdot IS_{\alpha }(F,y)\right),$$

where *w _k_* is a weight for every interval. Usually, $w_{k}=\frac{\alpha_{k}}{2}$ and *w*_0_ = 0.5.

The WIS is a proper scoring rule yielding non-negative values, with smaller values implying better performance. A forecaster, in expectation, optimises their score by providing a predictive distribution *F* that is equal to the data-generating distribution *G*, and is therefore incentivised to report their true belief. The WIS can be understood as an approximation of the continuous ranked probability score (CRPS, Gneiting *et al.* ^24^) for forecasts in a quantile-based format. The CRPS, in turn, represents a generalisation of the absolute error to predictive distributions. The WIS can be decomposed into three separate penalty components (corresponding to the three terms in the definition of the interval score): forecast dispersion (i.e., uncertainty of forecasts), overprediction and underprediction.

Bosse *et al.* ^25^ recently suggested to transform forecasts and observations using the natural logarithm prior to applying the WIS to better reflect the exponential nature of the underlying disease process. We, therefore, also compute WIS values after transforming all forecasts and observations using the function *f* : *x →* log( *x* + 1). In the following, we refer to WIS scores obtained without a transformation as "scores on the natural scale", and WIS values obtained after log-transforming forecasts and observations as "scores on the log scale". To make scores easier to interpret, we report relative WIS scores, where the average score for a given model was divided by the average score for the European Forecast Hub ensemble ("EuroCOVIDhub-ensemble"). In addition, we computed ranks based on WIS values.

In order to measure probabilistic calibration ^24^, we used the empirical coverage of all central 50% and 90% prediction intervals. Empirical coverage refers to the percentage of observations falling inside any given central prediction interval (e.g., the cumulative percentage of observed values that fall inside all central 50% prediction intervals).

If not otherwise stated, we present results for two-week-ahead forecasts, following the practice adopted by the COVID-19 Forecast Hubs, which found predictive performance to be poor and unreliable beyond this horizon ^1, 9, 11^. We analysed all forecasts stratified by forecast target (cases or deaths), forecast horizon, and forecast approach. We compared the performance of the direct vs. *R _t_* forecasting approach using instances where we had both a direct forecasts and an *R _t_* forecast from the same person.

For self-reported "experts" and "non-experts", a simple comparison of scores would be confounded by individual differences in participation and the timing of individual forecasts. We therefore compared the performance of self-reported "experts" vs. "non-experts" by creating and evaluating two modified median ensembles, one including only "experts" and the other only "non-experts".

Forecasts were evaluated using the scoringutils ^26^ package in R. All code and data used for this analysis, including individual-level forecasting data is available at https://github.com/epiforecasts/uk-crowd-forecasting-challenge. All code used to submit the forecasts to the European Forecast Hub is available at https://github.com/epiforecasts/europe-covid-forecast.

### Ethics statement

This study has been approved by the London School of Hygiene & Tropical Medicine Research Ethics Committee (reference number 22290). Consent from participants was obtained in written form.

## Results

### Crowd forecast participation

A total number of 90 participants submitted forecasts (more precisely, forecasts were submitted from 90 different accounts, some of them anonymous). Out of 90 participants, 21 self-identified as "experts", i.e., stated they had professional experience in infectious disease modelling or a related field.

The median number of unique participants in any given week was 17, the minimum was 6 and the maximum was 51. This was higher than the number of participants in 3 (which had a median number of 6, a minimum of 2, and a maximum 10). With respect to the number of submissions from an individual participant, we observed similar patterns as 3: An individual forecaster participated on average in 2.6 weeks out of 13. The median number of submissions from a single individual was one, meaning that similar to 3 most forecasters dropped out after their first submission. Only five participants submitted a forecast in ten or more weeks and only two submitted a forecast in all thirteen weeks, one of whom is an author on this study (S. Abbott). The number of direct forecasts (median: 13 for cases and 12 for deaths) was higher than the number of *R _t_* forecasts (median: 6 for both cases and deaths) in all weeks (see Figure 1A). The median number of "non-experts" (11 for cases, 10 for deaths) was higher than the median number of "experts" (8 for cases and deaths) (see Figure 1B).

### Case forecasts

**Figure 2.**
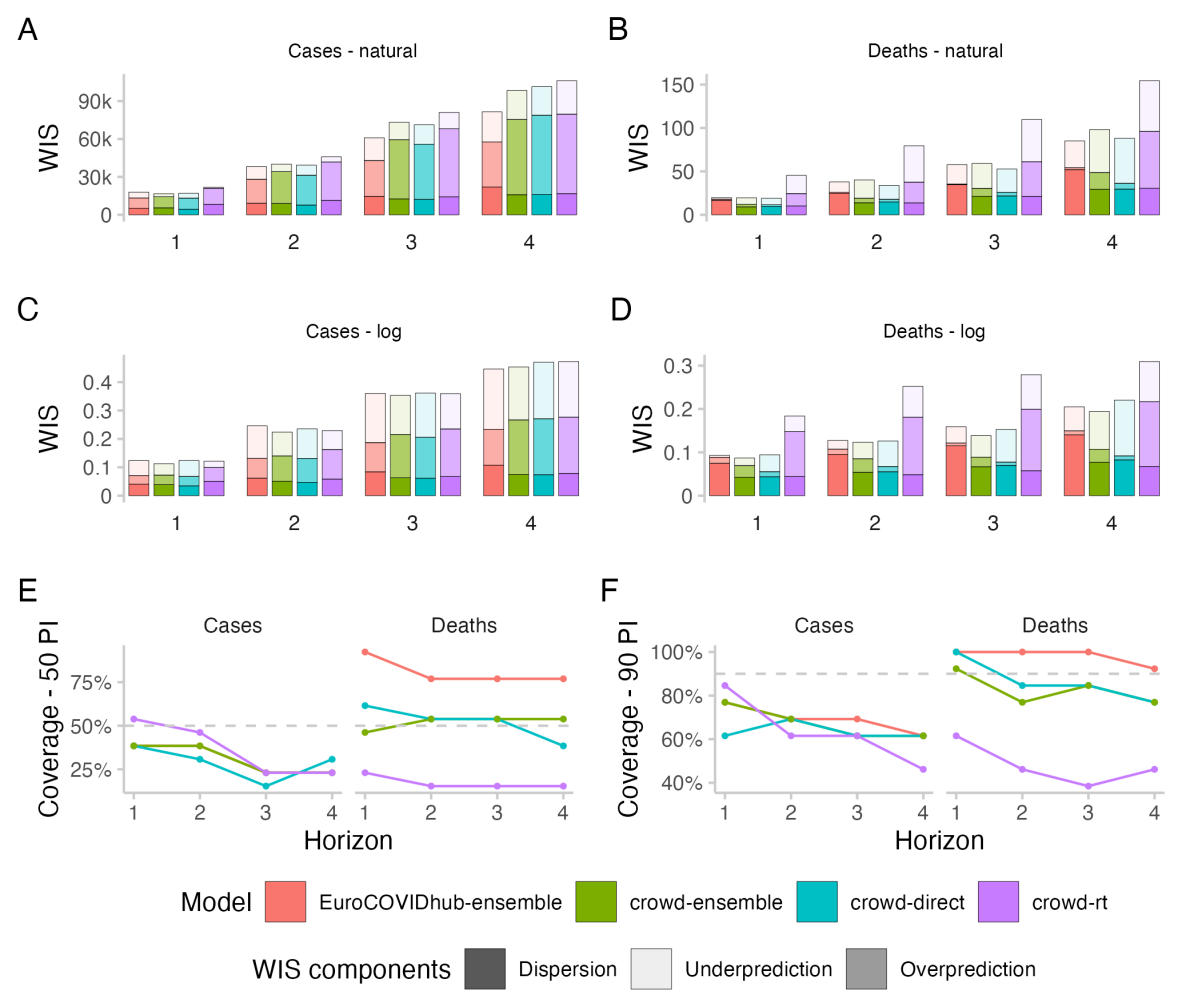
Predictive performance across forecast horizons. **A**– **D**: WIS stratified by forecast horizon for cases and deaths on the natural and log scale. **E**, **F**: Empirical coverage of the 50% and 90% prediction intervals stratified by forecast horizon and target type.

All forecasting approaches exhibited underdispersion when predicting cases, meaning that forecasts on average were too narrow and not uncertain enough. Empirical coverage for case forecasts was below nominal coverage for all forecasting approaches for forecasts more than one week into the future (see Figure 2E,F). For 50% prediction intervals, empirical coverage was worst for the direct crowd forecasts (0.31), best for the *R _t_* forecasts (0.46) and in between for the Hub ensemble and the crowd ensemble (both 0.38, see Table 1). For 90% prediction intervals, coverage was worst for the *R _t_* forecasts (0.62) and slightly better for the other approaches (all 0.69). Coverage for all forecasts deteriorated further with increasing forecast horizon (see Figure 2E,F).

**Table 1.** Performance for two-week-ahead forecasts. Values have been cut to three significant digits and rounded.

Model	Target	WIS - natural			WIS - log scale			Coverage 50%	Coverage 90%
		abs.	rel.	sd	abs.	rel.	sd		
EuroCOVIDhub-ensemble	Cases	38.2k	1	55.6k	0.25	1	0.22	0.38	0.69
crowd-ensemble	Cases	40.1k	1.05	69.4k	0.22	0.91	0.25	0.38	0.69
crowd-direct	Cases	39.3k	1.03	67k	0.23	0.96	0.27	0.31	0.69
crowd-rt	Cases	45.9k	1.2	74.7k	0.23	0.93	0.24	0.46	0.62
EuroCOVIDhub-ensemble	Deaths	37.9	1	26.9	0.13	1	0.04	0.77	1
crowd-ensemble	Deaths	40.2	1.06	41.5	0.12	0.97	0.07	0.54	0.77
crowd-direct	Deaths	33.9	0.89	30.6	0.13	0.99	0.08	0.54	0.85
crowd-rt	Deaths	79.5	2.1	72.7	0.25	1.98	0.13	0.15	0.46

**Figure 3.**
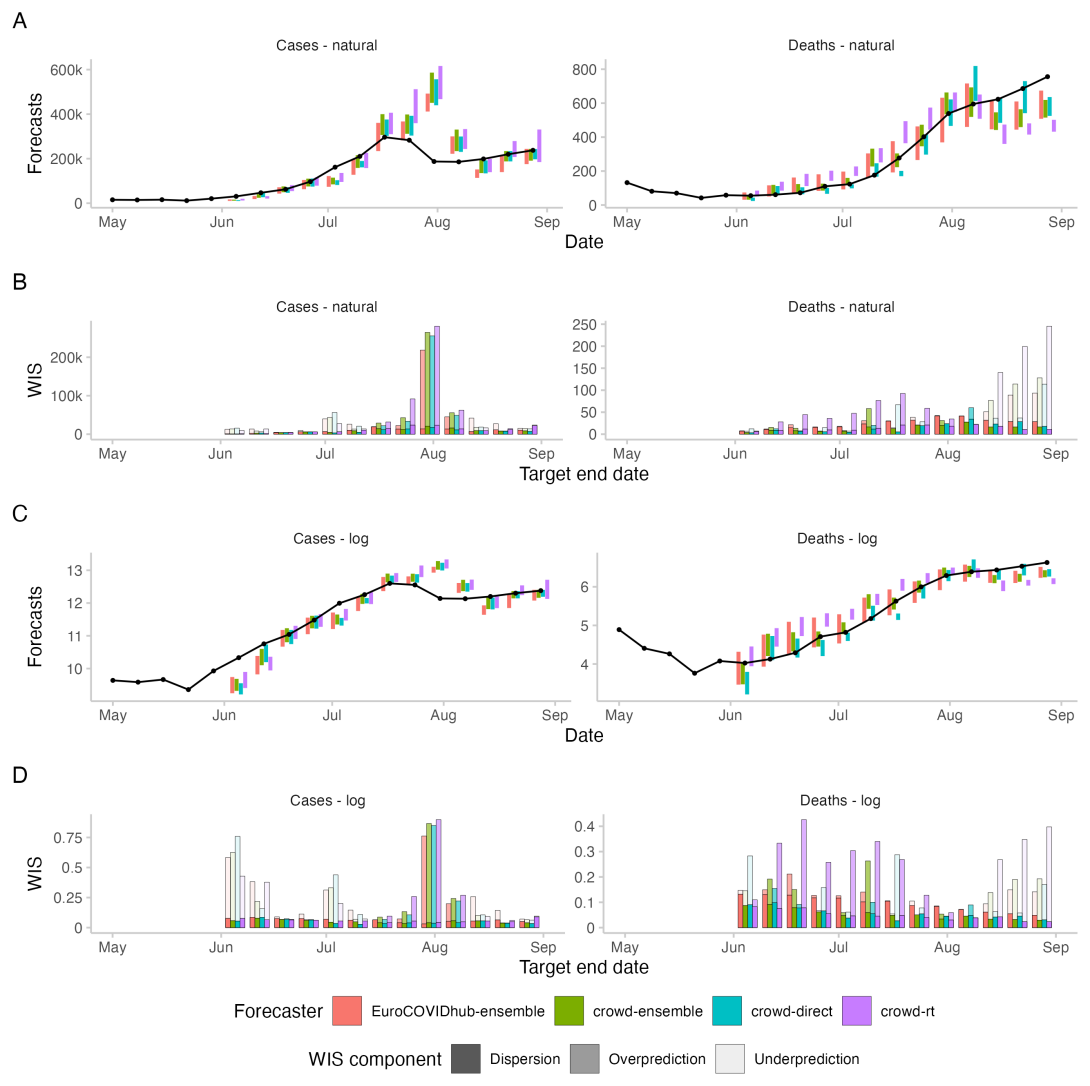
Forecasts and corresponding WIS for 2-week ahead forecasts of cases and deaths from COVID-19 in the UK. **A**: 50% prediction intervals (coloured bars) and observed values (black line and points) for cases and deaths on the natural scale. **B**: Corresponding WIS values, decomposed into dispersion, overprediction and underprediction. **C**: 50% prediction intervals on the log scale, i.e., after applying the natural logarithm to all forecasts and observations. **D**: Corresponding WIS on the log scale, i.e., the WIS applied to the log-transformed forecasts and observations.

In terms of WIS on the log scale, all human forecasting approaches outperformed the Forecast Hub ensemble for two week ahead forecasts of cases (see Figure 2). WIS values relative to the Hub ensemble (=1) were 0.91 for the combined crowd ensemble, 0.96 for the direct crowd forecasts and 0.93 for the *R _t_* forecasts (see Table 1). In contrast, in terms of WIS on the natural scale, the Hub ensemble outperformed all human forecasting approaches. Relative WIS values on the natural scale for two week ahead forecasts were 1.05 for the combined crowd ensemble, 1.03 for the direct crowd forecasts and 1.2 for the *R _t_* forecasts. The discrepancy between performance on the log and natural scale can be attributed to case forecasts from the Hub ensemble tending to be lower than forecasts from human judgement approaches (see Figure 3). On the natural scale, this resulted in smaller overprediction penalties, putting it ahead of human forecasts (see Figure 2A,C). On the log scale, however, it led to large penalties for underprediction.

Performance of the Hub ensemble relative to the human forecasting approaches improved with increasing forecast horizon (see Figure 2). For a four-week-ahead forecast horizon, the Hub ensemble outperformed all other approaches both on the log scale (rel. WIS values the human forecasts of 1.02, 1.05, 1.06) and on the natural scale (rel. WIS values of 1.21, 1.25, 1.3) (compare Table 1 in the Supplementary Information ^12^).

**Figure 4.**
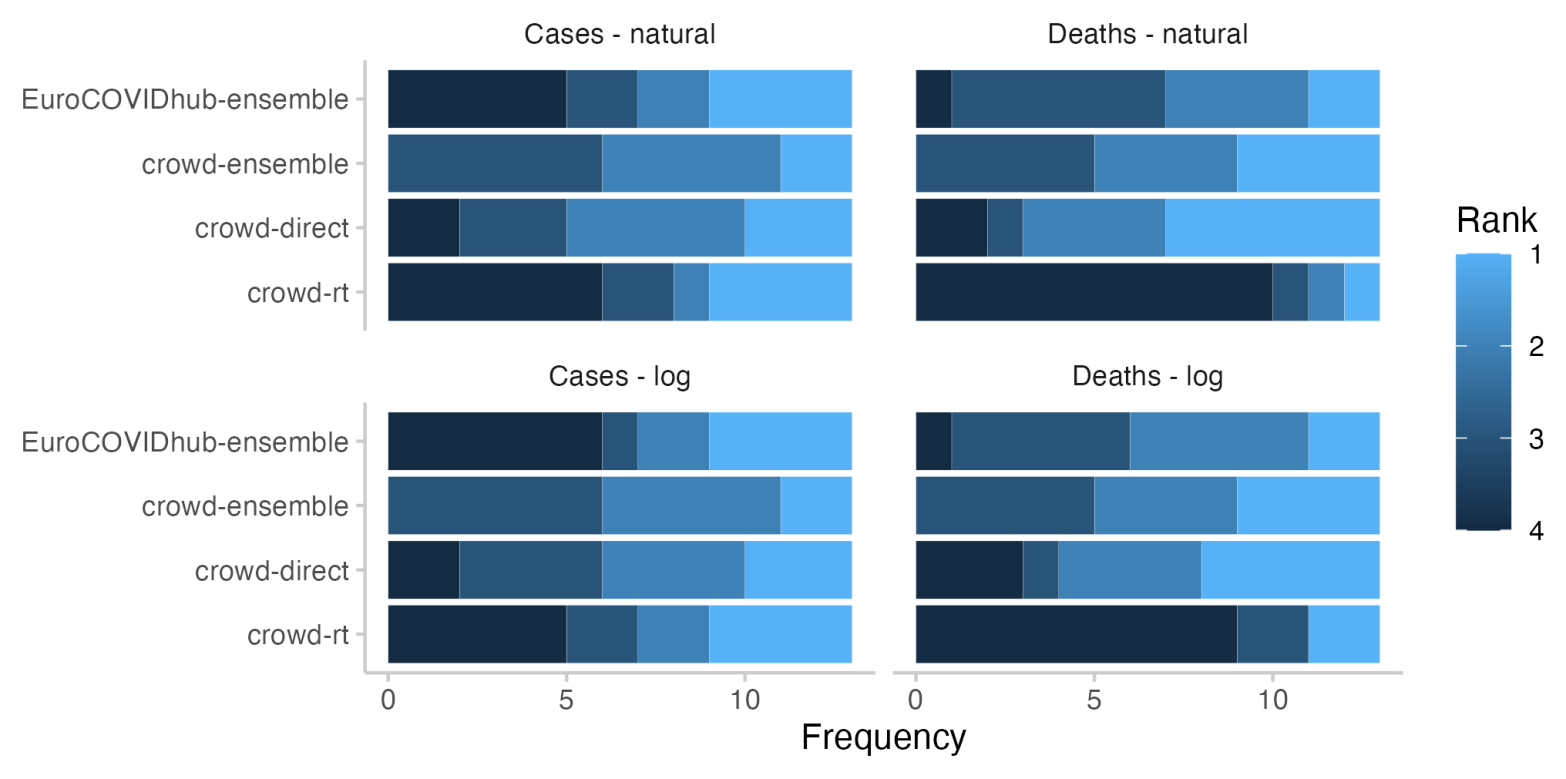
Ranks for all forecasting approaches for two week ahead forecasts. Colours indicate how often (out of 13 forecasts) a given approach got 1st, 2nd, 3rd, or 4th rank.

In terms of relative model ranks for two week ahead forecasts, the Hub ensemble and the *R _t_* forecast showed a higher variance than the combined crowd ensemble and the direct forecasts (See Figure 4), despite forecasts being about the same or more dispersed (see Figure 2). Both the Hub ensemble and the *R _t_* forecast were more often in first place than other approaches (4 times each, both on the log and on the natural scale). However, they were also most often in the last place (Hub ensemble: 6 on the log scale and 5 on the natural scale, *R _t_* : 5 on the log scale and 6 on the natural scale). The direct forecasts placed relatively equally in places 1-4. The crowd ensemble never placed fourth, but also had the lowest number of first places (2, both on the log and the natural scale). Aggregated model ranks only changed marginally when switching between the log and the natural scale (see Figure 4).

When comparing WIS values on the log scale with those on the natural scale, scores were more equally distributed across the study period on the log scale and more weight was given to forecasts in June and July which underpredicted the extent to which case number would rise (see Figure 3). On the natural scale, the WIS as a measure of the absolute distance between forecast and observation increased or decreased with the magnitude of the forecast target ^23, 25^. Average scores were therefore dominated by performance around the peak when cases were highest, in particular by forecasts made on the 19th of July for the 31st of July (see Figure 3). For all forecasting approaches, overprediction was the largest contributor to overall scores (see Figure 2A). On the log scale, underprediction played a larger role (see Figure 2C). Switching between scores on the log and on the natural scale had the strongest effect on the *R _t_* forecasts, which had a relative WIS value of 0.96 on the log scale and 1.2 on the natural scale. The *R _t_* forecasts tended to be higher than both the direct forecasts and the Forecast Hub ensemble, especially around the peak, leading to high scores on the natural scale, but not on the log scale.

### Death forecasts

All forecasting approaches except the *R _t_* forecasts showed higher empirical coverage for deaths than for cases (see Figure 2). Forecasts from the Hub ensemble generally tended to be wider than the human forecasts (see Figure 3 and Figure 2B,D). For 50% prediction intervals, the Hub ensemble exceeded the nominal coverage noticeably (0.77) (see Table 1). *R _t_* forecasts failed to get close to nominal coverage (0.15), while the combined crowd ensemble and the direct forecasts had empirical coverage close to nominal coverage (both 0.54). For 90% prediction intervals, the Hub ensemble again exceeded nominal coverage and covered all observations (1) while the *R _t_* forecasts again failed to get close to nominal coverage (0.46). The crowd ensemble exhibited some underdispersion (0.77) while the direct forcecasts almost reached nominal coverage for two week ahead forecasts of deaths (0.85).

In terms of WIS on the log scale for two week ahead predictions of deaths, the combined crowd ensemble (0.97) and the direct crowd forecasts (0.99) were marginally ahead of the Hub ensemble, while the *R _t_* forecasts performed noticeably worse (1.98) (see Figure 2D and Table 1). For the Hub ensemble, the dispersion component played by far the largest role, while this was less the case for the human forecasts, which got higher penalties from both over- and underprediction. Combining the *R _t_* forecasts and the direct forecasts led to an ensemble that performed better than either of them alone on the log scale despite the poor overall performance of the *R _t_* forecasts. In terms of WIS on the natural scale, only the direct forecasts (0.89) performed better for two week ahead death predictions than the Hub ensemble, while the combined crowd ensemble performed slightly worse (1.06) and the *R _t_* forecasts again noticeably worse (2.1).

In terms of relative model ranks for two week ahead death forecasts, the *R _t_* forecasts took the fourth place most often (9 on the log scale and 10 on the natural scale), while the direct forecasts placed first most often (5 on the log scale and 6 on the natural scale, see Figure 4). Again, the crowd ensemble never placed fourth.

When comparing scores on the log and on the natural scale, scores on the log scale were again more evenly distributed across the study period. On the natural scale, high scores were concentrated around the end of the study period, when death incidences were highest (see Figure 3).

### *R _t_* forecasts

**Figure 5.**
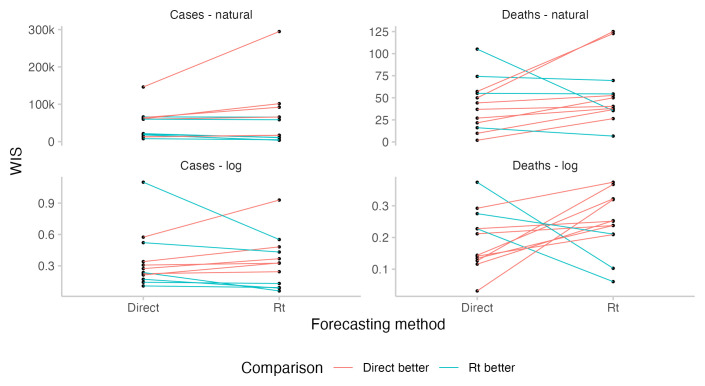
Comparison of predictive performance of individual forecasters using either the direct forecasting or *R _t_* interface. Comparisons are based only on those instances where forecasters have submitted a prediction using both interfaces. The absolute level for a given forecaster relative to others is not meaningful as forecasters differ in the amounts of forecasts they have submitted and when.

For cases, where participants could observe the case forecast implied by their *R _t_* forecast, predictive performance was similar between corresponding direct and *R _t_* forecasts for most forecasters who had submitted both (see Figure 5). For deaths, where forecasters could not see the incidence forecast implied by their *R _t_* forecast or manually adjust the case fatality rate, performance of the *R _t_* forecasts was significantly worse. From June to the end of July, *R _t_* forecasts overpredicted deaths and were noticeable higher than other forecasts, whereas in August, *R _t_* forecasts underpredicted deaths and were substantially lower than other forecasts (see Figure 3). In particular, *R _t_* forecasts for deaths were worse than the corresponding direct death forecasts for most forecasters (see Figure 5). Changing from the direct forecasting method to *R _t_* forecasting for cases tended to improve scores for better forecasters and decrease scores for worse forecasters, although sample sizes and the size of the observed effect are both small.

Combining direct crowd forecasts and *R _t_* forecasts improved performance on the log scale compared to both direct and *R _t_* forecasts alone across all horizons and target types. This was not the case on the natural scale, where direct forecasts performed better than the *R _t_* and the direct forecasts for both cases and deaths across most horizons. Only for case forecasts four weeks ahead on the natural scale was the combined ensemble better than the direct forecasts. However, even on the natural scale, performance of the combined ensemble was better than the average of the WIS of direct and *R _t_* forecasts.

### Experts and non-experts

**Figure 6.**
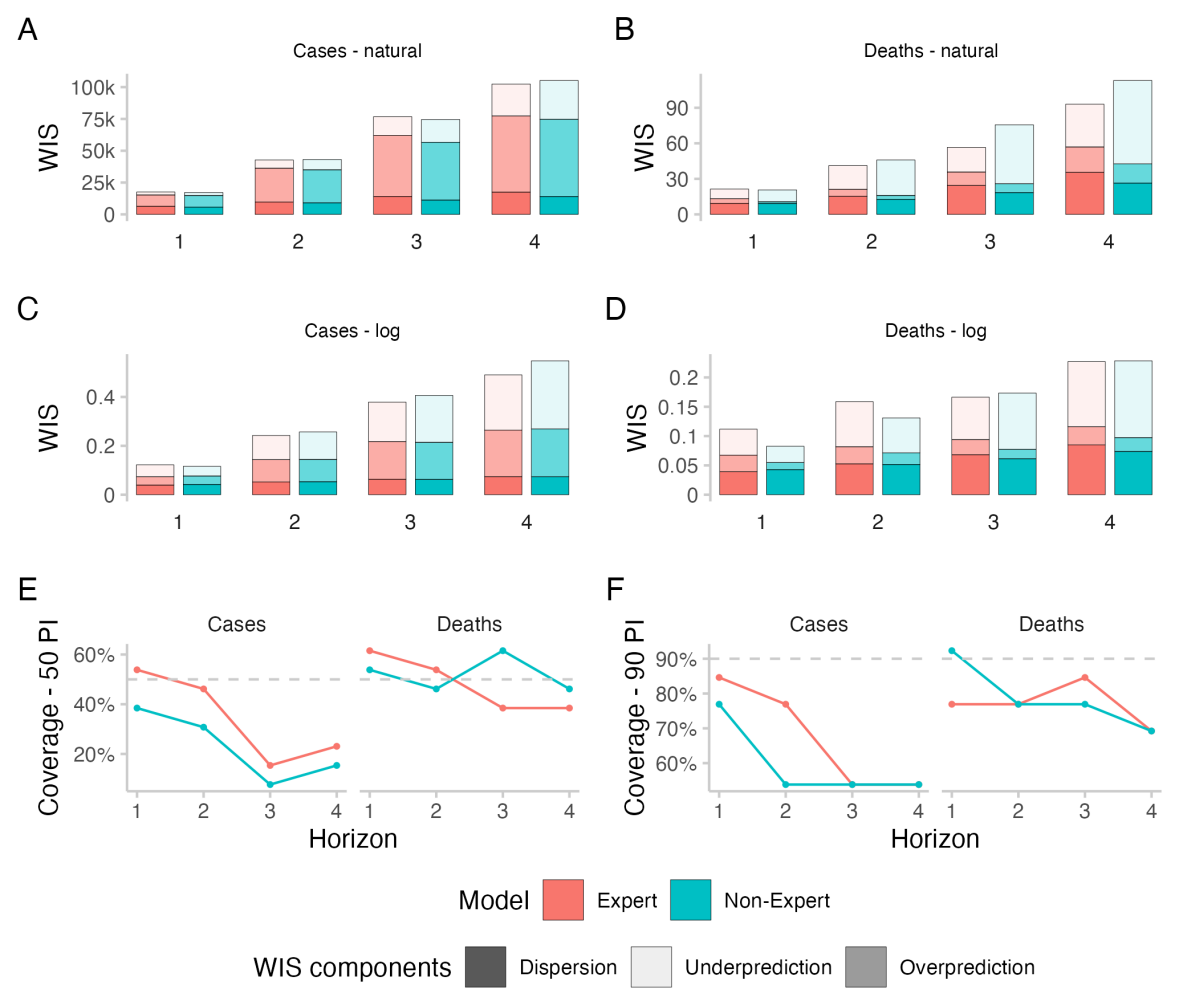
Predictive performance of self-reported "experts" and "non-experts" across forecast horizons. Forecasts from "experts" and "non-experts" were combined to two separate median ensembles, including both direct and *R _t_* forecasts. **A**– **D**: WIS stratified by forecast horizon for cases and deaths on the natural and log scale. **E**, **F**: Empirical coverage of the 50% and 90% prediction intervals stratified by forecast horizon and target type.

A median ensemble of two week ahead forecasts restricted to only those made by either "experts" or "non-experts" (determined based on self-reported experience in infectious disease modelling or a related field) performed worse than the combined crowd example, both for cases and deaths and both on the log scale and on the natural scale (see Figure 6 and Table 2 and Figure 1B for a visualisation of participation). The median number of "non-experts" was 11 for cases and 10 for deaths, which was higher than the median number of "experts", which was 8 for cases and deaths.

**Table 2.** Performance for two-week-ahead forecasts of experts and non-experts. Values have been cut to three significant digits and rounded.

Model	Target	WIS - natural			WIS - log scale			Coverage 50%	Coverage 90%
		abs.	rel.	sd	abs.	rel.	sd		
crowd-ensemble	Cases	40.1k	1	69.4k	0.22	1	0.25	0.38	0.69
Expert	Cases	42.7k	1.06	74.9k	0.24	1.08	0.28	0.46	0.77
Non-Expert	Cases	43.1k	1.07	67k	0.26	1.14	0.25	0.31	0.54
crowd-ensemble	Deaths	40.2	1	41.5	0.12	1	0.07	0.54	0.77
Expert	Deaths	41.2	1.03	41.8	0.16	1.29	0.15	0.54	0.77
Non-Expert	Deaths	45.9	1.14	56.8	0.13	1.06	0.08	0.46	0.77

When comparing two week ahead forecasts from "experts" and "non-experts", the ensemble of "experts" was better calibrated (see Figure 6). For cases, "experts" achieved better scores than "non-experts" both on the log and on the natural scale. WIS values *relative to the combined crowd ensemble* were 1.08 for "experts" and 1.14 for "non-experts" on the log scale and 1.06 for "experts" and 1.07 for "non-experts" on the natural scale (see Table 2). For deaths, "experts" performed worse than "non-experts" in terms of WIS on the log scale (WIS relative to the combined crowd ensemble: 1.29 vs. 1.06), but better on the natural scale (1.03 vs. 1.14). Both the "expert"- and the "non-expert"-ensemble had similar proportions of *R _t_* forecasts (mean of 32% for "experts" and 32.2% for "non-experts" across cases and deaths together).

For four weeks ahead forecasts of cases, the combined ensemble outperformed both "experts" and "non-experts" on the log scale as well as on the natural scale. "Experts" performed better than "non-experts" both on the log scale (WIS value relative to the combined crowd ensemble of 1.08 for "experts" vs. 1.21 for "non-experts") and on the natural scale (1.04 vs. 1.07). For four week ahead forecasts of deaths, "Experts" performed better than "Non-experts" on the log scale (1.17 vs. 1.18) as well as on the natural scale (0.95 vs. 1.15).

## Discussion

In this paper, we presented a follow-up study to 3, analysing human judgement forecasts of cases of and deaths from COVID-19 in the United Kingdom submitted to the European COVID-19 Forecast Hub between the 24th of May and the 16th of August 2021. Human judgement forecasts were generated using two different forecasting approaches, a) direct forecasts of cases and deaths and b) forecasts of the effective reproduction number *R _t_*, which were based on estimates from an open source effective reproduction number estimation model and also relied on this model, along with a second model relating cases and deaths from the same source, to simulate reported cases and deaths.

Just like Bosse *et al.* ^3^ and Farrow *et al.* ^7^, this study struggled to retain a large number of participants. Focused public outreach efforts such as creating a dedicated website, announcing an official tournament, providing a public leaderboard, sending weekly emails with details on past performance and weekly announcements on Twitter, did noticeably increase participation compared to the previous study in Germany and Poland. Nevertheless, retaining participants beyond the initial recruitment proved challenging, and most forecasters only submitted a single forecast. McAndrew *et al.* ^5^ had a higher number of participants, suggesting that making use of existing forecasting platforms that have access to a large existing user base and greater resources may be helpful in recruiting a larger number of participants, though these platforms lack the flexibility and software tooling to run a novel study of this kind in real-time as things stand.

In line with results from previous work ^3, 11^, we found almost all forecasts for cases to be underdispersed (i.e., too narrow/overconfident). Empirical coverage for death forecasts was higher than the corresponding coverage for cases for all forecasting approaches except the *R _t_* forecasts.

For forecasts of cases two weeks ahead, performance of the human judgement forecasts was better than the European Forecast Hub ensemble in terms of WIS on the log scale, and worse in terms of WIS on the natural scale. This was linked to a tendency of the Hub ensemble to make lower case predictions, which led to lower overprediction penalties on the natural scale, but noticeably higher underprediction penalties on the log scale. For forecasts of deaths two weeks ahead, direct human forecasts and the combined crowd ensemble performed better than the Hub ensemble on the log scale. On the natural scale, the combined crowd ensemble performed worse than the Hub ensemble, while the direct crowd forecasts still performed better. *R _t_* forecasts for deaths performed noticeably worse than all other approaches both on the log and on the natural scale.

In their original study, conducted in Germany and Poland, Bosse *et al.* ^3^ found that humans outperformed an ensemble of computational models when predicting cases, but not when predicting deaths. They hypothesised that computational models might have an advantage over human forecasters when predicting deaths, benefiting from the ability to model the delays and epidemiological relationships between different leading and lagged indicators. McAndrew *et al.* ^5^ similarly found in their study that humans performed comparably to an ensemble of computational models for cases, but not for predictions of deaths of COVID-19. Results in our study do not directly support this pattern, but given the low number of observations also do not provide strong evidence against it. In this study, the combined crowd ensemble performed better than the Hub ensemble on both cases and deaths on the log scale, and worse on the natural scale. Direct forecasts, which would be most comparable to the forecasts in 3, performed worse than the Hub ensemble on cases and better on deaths. During the study period, the case fatality ratio (CFR) changed quite quickly. This was related to the rise of the Delta variant in the UK in the beginning of May with its higher case fatality rate ^27^. In addition, the age distribution of cases changed over the course of the study period in Summer 2021, in parts related to the European Football Championship ^28^. One possible hypothesis for the relatively good performance of human forecasts for deaths compared to previous studies might be that some models submitted to the Forecast Hub may have been more negatively affected by the changes in CFR during the study period than human forecasters or have been slower to update. A confounding factor, when comparing results from this study and the one in Germany and Poland directly, is that we used a median ensemble to combine individual forecasts here, while the earlier study used a mean ensemble.

This study explored a novel method of forecasting infectious diseases that combines a human forecast of the estimated effective reproduction number *R _t_* with epidemiological modelling to map the *R _t_* forecast to a forecast of cases and deaths. One appeal of this approach is that the forecaster can directly forecast the generative process and how they believe it is affected by interventions and changes in behaviour. Computational modelling then takes care of dealing with details such as reporting delays, generation intervals, day of the week periodicity, and the relationship between different indicators. This could help reduce cognitive load, and make it easier to synthesise various sources in information into a single forecast, at least for forecasters who have an intuitive understanding of *R _t_*. Though we note all of these modelling steps and the construction of the model itself requires the human constructing the model to make assumptions. Anecdotally, forecasters familiar to the authors reported high satisfaction with the forecasting experience. In our study, *R _t_* forecasts of cases were comparable to direct forecasts, with a tendency for good forecasters to improve when using the *R _t_* method and worse forecasters to deteriorate even more. Sample sizes, however, were very low. Given that forecasters could simulate cases in the app, it is also possible that forecasters were in fact directly forecasting cases. *R _t_* forecasts of deaths (which forecasters could not see in the app) were noticeably worse than direct forecasts of deaths. The computational model underlying our *R _t_* forecasts of deaths estimated a constant CFR and delay distribution using the last 4 weeks of data, therefore updating relatively slowly to new circumstances and the CFR was assumed to be constant over the four week forecast horizon. However, as mentioned before, the CFR evolved during the study period. Forecasters had no way of inspecting the death forecast implied by their *R _t_* forecast and also had no way to adjust the CFR manually, likely impacting forecast accuracy. Giving human forecasters the ability to adjust the CFR and other model parameters would have increased complexity of the interface, but would have solved issues with the assumptions of the underlying model. Alternatively, a more complex model could have been used which allowed for time-varying CFR estimates and forecast these changes over the forecast horizon though this approach may still have struggled to cope with the rapid changes observed during the study period. Another important limitation is that we didn’t have full sample trajectories of the *R _t_*-values predicted by forecasters. Rather, trajectories had to be constructed based on the distributions provided for the different forecast horizons, which likely negatively affected forecasts. One potential way to disentangle the effect of the convolution model from the *R _t_* forecasts would have been to use the human forecasts for cases as an input to the second computational model, which could then have simulated deaths. Future work could expose forecasters to different combinations of these options with the aim of separating effects of the user interface from ones related to the structure of the underlying computational model.

Combining forecasts from "experts" and "non-experts" led to better performance for forecasts two weeks ahead for cases as well as deaths, and both on the log scale and on the natural scale. Combining direct forecasts and *R _t_* forecasts led to better performance on the log scale, but not on the natural scale. This suggests that combining different forecasts can be beneficial in many instances, although there may be differences in terms of WIS on the log and the natural scale. In particular, WIS values on the natural scale may be more susceptible to models that would tend to overshoot and miss the peak, while WIS on the log scale may be more affected by models that underpredict and miss upswings ^25^.

Past studies of expert forecasts of COVID-19 ^6^ had found predictions from experts to outperform those of non-experts. In our study, an ensemble of self-reported "experts" outperformed an ensemble of "non-experts" when forecasting cases two weeks ahead, both on the log scale and on the natural scale. When forecasting deaths two weeks ahead, "experts" performed worse than "non-experts" on the log scale, but better on the natural scale. Forecasts for "experts" tended to be better calibrated than non-experts. However results should be taken with care considering relatively low sample sizes (median of 11 "non-experts" for cases and 10 for deaths, median of 8 "experts" for cases and deaths) and given that expert status was self-reported. Furthermore, we only asked for professional involvement in a field related to infectious disease modelling, not specifically for familiarity with modelling of COVID-19 in the UK, and only offered participants a binary choice. However, as we used ad-hoc recruitment in our networks many of these self-identified experts are likely to be infectious disease modellers.

Overall, results of our study should be taken with caution due to several important limitations. Firstly, our study was restricted to one location and to a relatively short period of thirteen weeks. Secondly, there were many confounding factors that likely influence results. These include the fact that different participants made forecasts at different points in time (with the median forecaster only submitting a single forecast) and that subgroups of interest (e.g. "experts", or *R _t_* forecasts) had different numbers of forecasters. In most instances, differences in scores between forecast approaches were small compared to the variance of scores within a single approach. In addition, there were many researcher degrees of freedom that could influence findings, for example how individual forecasts were combined to create an ensemble. Results were influenced by choices made during the evaluation with, for example, some conclusions depending on forecast horizon and the transformation used prior to scoring. Highlighting this, prizes to the human forecasters were paid out based on the combined WIS on the log scale across all horizons and forecast targets. Had we chosen to instead measure WIS on the natural scale, or to forecast only cases and continue to score on the log scale, rankings and payouts would have been different.

## Conclusions

The results of our study are broadly consistent with previous studies on human judgement forecasting of COVID-19 and suggest that human crowd ensembles and an ensemble of computational models are able to produce forecasts of similar quality. One interpretation of these findings is that a mixed crowd of human forecaster can produce a viable alternative or complement to an ensemble of mathematical models created by experts. An altnerative interpretation is that an ensemble of automated models can produce forecasts over the course of several years that are on par with that of an engaged crowd of human forecasters. This study, and all previous studies, comparing human judgement forecasts and computational models only ran over short periods of time and the majority of them struggled with recruitment and upkeep. Meanwhile, COVID-19 Forecast Hubs have attracted continuous submissions for almost three years and were able to consistently provide forecasts of comparable quality.

Our findings do not suggest that humans are necessarily at a general disadvantage compared to computational models at predicting reported deaths, but evidence in both directions is limited and this is made particularly complex as our study took place during a period of time when CFR estimates were changing rapidly. Despite evaluations being public, it remains a challenge to properly incentivise contributors to Forecast Hubs to regularly update their forecasting methodology in order to maximise utility, predictive performance, or both. Combining human judgement and epidemiological modelling by mapping *R _t_* forecasts to case and death numbers has not yielded competitive forecasts for deaths in this study. However, we only presented a prototype of a forecasting approach, which, while having appealing properties, proved challenging to implement. Subsequent iterations and improvements could likely achieve better results. More research is required to obtain a better understanding of the role of subject matter expertise in infectious disease forecasting. Our results underline that it is difficult to evaluate forecast performance devoid of context that helps inform what a good or a bad forecast is. Different ways to look at the data let different forecasts appear better or worse. Forecast evaluation therefore either needs to be clearly informed by the needs of forecast consumers to determine what a good forecast is, or it needs a broad array of perspectives to provide a wholistic picture as we have attempted to present in this work. Furthermore, evaluating forecasts post-hoc leaves the researchers with many degrees of freedom to make decisions that affect which models look good and there is a risk of allowing for motivated reasoning. More emphasis should be put on measures that prevent this, e.g. by establishing common standards for evaluations, pre-registering studies, and making it a norm to display a variety of standard metrics.

## Data Availability

All data and code are available publicy under a MIT license under https://github.com/epiforecasts/uk-crowd-forecasting-challenge and https://doi.org/10.5281/zenodo.7897257. The data has been published separately here: https://doi.org/10.5281/zenodo.7897289. Supplementary Information ^12^ to this manuscript is available at https://doi. org/10.5281/zenodo.7897513.
