# Supplementary material for: Human judgement forecasting of COVID-19 in the UK
Source: Wellcome Open Res. 2024 Mar 21;8:416. Originally published 2023 Sep 19. [Version 2] doi: 10.12688/wellcomeopenres.19380.2 (PMC11009611; doi:10.12688/wellcomeopenres.19380.2)
Supplement: Peer review history

# Reviewer report: Reviewer response for version 2

**DOI:** 10.21956/wellcomeopenres.23498.r77351

**Published:** 2024-04-11

**Authors:** Daniel J. McDonald

**Affiliations:** 1 Department of Statistics, The University of British Columbia, Vancouver, British Columbia, Canada

The authors responded carefully and effectively to the comments in my previous report. I have no further reservations.

Is the work clearly and accurately presented and does it cite the current literature?

Yes

If applicable, is the statistical analysis and its interpretation appropriate?

Yes

Are all the source data underlying the results available to ensure full reproducibility?

Yes

Is the study design appropriate and is the work technically sound?

Yes

Are the conclusions drawn adequately supported by the results?

Yes

Are sufficient details of methods and analysis provided to allow replication by others?

Yes

Reviewer Expertise:

Epidemic forecasting; Statistics

I confirm that I have read this submission and believe that I have an appropriate level of expertise to confirm that it is of an acceptable scientific standard.

# Reviewer report: Reviewer response for version 1

**DOI:** 10.21956/wellcomeopenres.21467.r73036

**Published:** 2024-02-20

**Authors:** Hongru Du

**Affiliations:** 1 Johns Hopkins University, Baltimore, Maryland, USA

 This manuscript extends the investigation presented in reference 3, focusing on applying human judgment forecasts for COVID-19 cases and deaths within the United Kingdom. These human-generated forecasts were provided in two distinct styles: a) direct predictions and b) Rt-based forecasts. These forecasts' findings were then compared to those from the EuroCOVIDhub-ensemble. The overall quality of the paper is commendable, with its content being coherent and its main points effectively communicated. Below are further comments:

1. The assumptions for the Rt forecast need a more precise description. Given that the reported cases represent merely a subset of all infections, estimating Rt based on these reported cases may not accurately reflect the true Rt value. It's essential to clarify how this limitation is addressed in the forecast model.
2. The benefits of human-based forecasting should be more prominently emphasized. I think the primary advantage of such forecasts lies in the human ability to understand a broader spectrum of information, particularly crucial insights that are difficult to quantify and input as numerical data. For instance, the period under review coincided with the spread of the Delta variant across Europe. Suppose experts were furnished with early official reports detailing the relative infectiousness or severity of the Delta variant. Could this have enhanced the ability of human forecasters to identify pivotal turning points, such as those occurring in early June? Exploring an alternative approach by providing human forecasters with more real-time, descriptive information, rather than solely relying on numerical data like test positivity and vaccination rates, can be an interesting extension of current work. 
3. One minor comment about Figures 2 E and F is what the grey dash line means and why the Ensember model is not showing in E for cases. 

Is the work clearly and accurately presented and does it cite the current literature?

Yes

If applicable, is the statistical analysis and its interpretation appropriate?

Yes

Are all the source data underlying the results available to ensure full reproducibility?

Yes

Is the study design appropriate and is the work technically sound?

Yes

Are the conclusions drawn adequately supported by the results?

Yes

Are sufficient details of methods and analysis provided to allow replication by others?

Yes

Reviewer Expertise:

AI in public health, Infectious disease modeling

I confirm that I have read this submission and believe that I have an appropriate level of expertise to confirm that it is of an acceptable scientific standard.

# Reviewer report: Reviewer response for version 1

**DOI:** 10.21956/wellcomeopenres.21467.r67709

**Published:** 2023-10-17

**Authors:** Daniel J. McDonald

**Affiliations:** 1 Department of Statistics, The University of British Columbia, Vancouver, British Columbia, Canada

This manuscript describes the results of a crowd forecasting challenge conducted during the summer of 2021 in the United Kingdom. Participants were asked to predict Covid-19 cases and deaths in the UK during the period across a collection of forecast horizons and to provide uncertainty along with point predictions. The participants could (and were encouraged to) participate weekly, with a cash incentive.

 The results are somewhat mixed in terms of success of the study, with potentially the largest barrier being participant adherence (similar to related work). Nonetheless, participant submissions were (on average) of comparable accuracy to the EuroCOVIDhub-ensemble. The manuscript is well-written and provides a detailed and thorough discussion of the results, highlighting successes and drawbacks. It also serves as a strong template, illustrating current best-practices, for other work evaluating these sorts of forecasts. A few (minor) suggestions for improvement are listed below. 

1. The period of study, May-August 2021, deserves more context as well as a few paragraphs in the discussion (see also point 3 below). I would suggest, early on, including a figure (like SI.2) that shows the ground truth from the beginning of the pandemic, up to the end of the study period (or possibly to the present), highlighting the period of study. This would give the reader some context of what was happening during the study, along with some idea of the phenomena that the forecasters were basing their forecasts on. Importantly, as seen in Figure 3, both cases and deaths followed a general upward trajectory. This is important for a number of reasons: (a) possibly explains the over/underprediction at the beginning/end of the period; (b) possibly contrasts with conclusions that could be made during flat or declining periods or more balanced around a peak; (c) impacts the definition of "around the peak" at the end of the Case forecasts section; (d) impacts the relative difficulty of the forecast task; (e) informs some of the discussion of log vs natural scale performance.
2. One author is mentioned in the body as having participated (presumably as an expert), while another won the challenge (according to the Acknowledgements). How many of the authors participated in the challenge (and how many of the total submissions are they responsible for)? It is worth being more forthcoming about this as well as perhaps analyzing how this participation impacts the results.
3. The CFR discussion (in the Discussion) is relevant, but could be clarified. Some statements about "changes" should be supported more convincingly (is this from literature, some estimate not shown, etc). Additionally, it's assumption as constant for the Rt task (described in the Discussion) seems less important than the fact that the implied Death forecast wasn't shown (described in the Methods). I suspect that changing CFR is less important for the accuracy of the forecasts than the period of study, so these probably deserve at least equal treatment.
4. The comparison to the EuroCOVIDhub-ensemble is illustrative, but it would help to compare with the individual forecasters as well. Figure 4, for example, is not particularly illuminating, but perhaps with a larger sample of (almost certainly worse) forecasts, it would serve to further emphasize the relative accuracy of the human judgement forecasts.  Related, because these judgement forecasts were submitted to the Euro COVID hub, they are presumably included in the hub-ensemble? How might this impact your results about relative performance?
5. It is possibly described in Reference 3, but it would be interesting to examine the priming effects of the initially displayed “Baseline” forecast (reasonable to defer to future work). If the intervals were wider, would the judgement forecasts be better calibrated? If they sloped up would the forecasters be more likely to predict an increase? To some degree the Rt version is a different type of “priming” (as shown in SI.4).

Is the work clearly and accurately presented and does it cite the current literature?

Yes

If applicable, is the statistical analysis and its interpretation appropriate?

Yes

Are all the source data underlying the results available to ensure full reproducibility?

Yes

Is the study design appropriate and is the work technically sound?

Yes

Are the conclusions drawn adequately supported by the results?

Yes

Are sufficient details of methods and analysis provided to allow replication by others?

Yes

Reviewer Expertise:

Epidemic forecasting; Statistics

I confirm that I have read this submission and believe that I have an appropriate level of expertise to confirm that it is of an acceptable scientific standard, however I have significant reservations, as outlined above.
